# Exogenous Hormonal Application Regulates the Occurrence of Wheat Tillers by Changing Endogenous Hormones

**DOI:** 10.3389/fpls.2018.01886

**Published:** 2018-12-21

**Authors:** Tie Cai, Xiangping Meng, Xiaoli Liu, Tiening Liu, Hui Wang, Zhikuan Jia, Dongqing Yang, Xiaolong Ren

**Affiliations:** ^1^Key Laboratory of Crop Physi-Ecology and Tillage Science in North-Western Loess Plateau, College of Agronomy, Northwest A&F University, Yangling, China; ^2^State Key Laboratory of Crop Biology, College of Agronomy, Shandong Agricultural University, Tai’an, China

**Keywords:** wheat, hormonal, tiller, regulation, yield

## Abstract

Plant hormones play important roles in regulating the occurrence of crop tillers. However, little is known about the relationships and the underlying mechanisms between endogenous hormones and the occurrence of wheat tillers induced by exogenous hormones. In this study, two winter wheat cultivars, Xinong 979 and Xiaoyan 22, were used to investigate the effects of the exogenous application of indole-3-acetic acid (IAA) and zeatin (Z) on the occurrence of wheat tillers and investigate underlying mechanisms regulating the occurrence of tillers. The results showed that the application of IAA inhibited the occurrence of tillers, and external Z application promoted the occurrence rate of tillers under low nitrogen conditions. Further analysis of the results showed that exogenous IAA completely inhibited the growth of tiller buds, while exogenous Z significantly promoted the growth rate of tiller buds in low nitrogen conditions. Endogenous hormones exhibit important functions in regulating the growth of tiller buds, which contents were affected by exogenous hormones. Furthermore, according to the principal component analysis and correlation analysis, the growth of tiller buds was significantly positively correlated with the content of endogenous Z, whereas it was significantly negatively correlated with the ratios of endogenous IAA to endogenous Z (IAA:Z) and endogenous abscisic acid (ABA) to endogenous Z (ABA:Z). Moreover, no significant correlation was observed between the growth of the tiller buds and the endogenous IAA, endogenous gibberellins (GAs), and endogenous ABA content. These results suggested that Z played key roles in regulating the tiller occurrence, and exogenous hormones regulated the growth of wheat tiller buds via affecting the Z contents, thus regulating the occurrence of wheat tiller.

## Introduction

**FIGURE 1:**
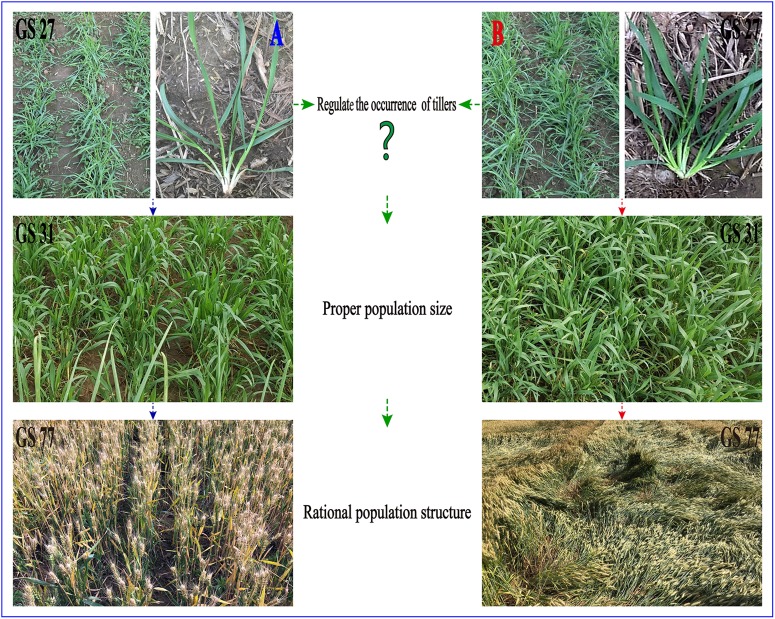
Difference in wheat population. **(A)** Too few surviving tillers leads to a smaller population, resulting in lower crop yields due to less spikes number per unit area. **(B)** Too many surviving tillers leads to a larger population, which may cause lodging in the late growth stage.

Wheat (*Triticum aestivum* L.) is one of the most essential crops worldwide (Yang et al., 2018). Population growth and social development necessitate a rigid demand for high-yield wheat, which at present, mainly relies on raising yield per unit area (Wei et al., 2016). Additionally, the yield potential of wheat can be dissected into four major components: plant number per unit area, spike number per plant, grain number per spike, and grain weight (Yu, 2013). The number of tillers of an individual wheat plant, which determines the spike number per plant, is an important agronomic trait for grain production (Wang et al., 2016). Affected by environmental factors, the number of tillers is dynamic and adjustable (Cai et al., 2014). Tiller development is greatly inhibited by abiotic stressors, such as drought, low nitrogen, and low temperature, resulting in lower crop yields due to a smaller population (Mitchell et al., 2012) (Figure 1A). In contrast, too many surviving tillers owing to better environmental factors leads to a larger population, which may cause lodging in the late growth stage because of deterioration of the stem quality (Zheng et al., 2017) (Figure 1B). Therefore, it is essential to construct a rational population structure for wheat to enhance yield potential and avoid lodging.

It is key to regulate the occurrence of tillers to obtain the proper number of tillers within a wheat population. The occurrence of tillers generally involves two developmental stages: the formation of axillary meristems in the leaf axil and the growth of tiller buds (Li et al., 2003). Of the two developmental stages, the former is mainly determined by genetics (Hyles et al., 2017), while the growth of tiller buds is more sensitive to environmental elements. Additionally, external treatments have a significant effect on the growth of buds (Assuero and Tognetti, 2010). The occurrence of tillers is usually marked by the first complete leaf of the tiller protruding out of the coleoptile or tiller sheath (Yu, 2013), indicating that the growth of tiller buds directly determines the occurrence of tillers. Therefore, promoting or inhibiting the growth of a tiller bud in a specific position can regulate the occurrence of this tiller. Currently, there is no effective technical approach for the direct control of the growth of wheat tiller buds.

The growth of tiller buds is regulated by a variety of hormones (Lin et al., 2016), of which indole-3-acetic acid (IAA) is the earliest discovered class of plant hormones that are synthesized at the apices and are transported down the polar base to inhibit the growth of lateral buds (Wang et al., 2006). External application of naphthyl acetic acid (NAA, a synthetic auxin) completely inhibits the growth of tiller buds in rice (Liu et al., 2011a). In contrast to IAA, cytokinins (CTKs) have a stimulatory effect on lateral bud outgrowth (Kariali and Mohapatra, 2007). Previous studies have shown that higher zeatin nucleosides (ZR, a cytokinin) can promote the sprouting of tiller buds in maize (Wang et al., 2012b), and increasing the CTK content in tiller nodes can accelerate the growth of rice tiller buds (Liu et al., 2009). In addition to the regulation of IAA and CTK, other hormones also significantly affect the growth of lateral buds. The application of gibberellic acid (GAs) markedly inhibits the occurrence of crop tillers (Zhang, 1997). Consistent with this result, our previous study also showed that spraying GA_3_ can significantly inhibit the growth of tiller buds. Moreover, external application of abscisic acid (ABA) slightly slows the growth rate of rice tiller buds (Liu et al., 2011a). Further studies have found that exogenous ABA affected the growth of tiller buds by increasing the endogenous ABA content in tiller nodes (Cai et al., 2013). Therefore, external hormone application has been shown to be an effective measure to regulate the growth of tiller buds.

Similarities exist among different crops in physiological characteristics and growth regulation measures; however, there are also marked differences as well. Several relatively systematic studies have been conducted on the relationship between hormones and the occurrence of tillers in rice (Liu et al., 2011a; Xu et al., 2015) and maize (Wang et al., 2012a,b). However, there is limited information available about hormones and the occurrence of wheat tillers. Furthermore, some experiments have shown that IAA applied to the top of plants cannot enter lateral buds (Booker et al., 2003), indicating that IAA does not directly inhibit the growth of lateral buds, and there may be secondary messengers that transmit IAA signals to lateral buds. At present, some hormones have been confirmed as secondary messengers such as CTKs (Domagalska and Leyser, 2011). Moreover, studies have also suggested that the function of GAs and ABA do not directly affect lateral buds but might change the content of other hormones to affect the growth of tiller buds (Morris and Choonseok, 2006). Therefore, the role of hormones in regulating the growth of tiller buds needs to be further clarified.

In order to address these limitations of previous research (Currently, ➀ there is no effective technical approach for the direct control of wheat tiller occurrence at present; ➁ there is limited information available about hormones and the occurrence of wheat tillers; ➂ there are different opinions on the role of endogenous hormones such as IAA, CTKs, GAs, ABA in the growth of tiller buds.), the objectives of the present study were to investigate the effect of exogenous IAA and Z on the occurrence of tillers in wheat and analyze the relationship between endogenous hormones and the growth of wheat tiller buds. A greater understanding of these relationships, both positive and negative, can provide a theoretical basis to improve regulatory techniques for the effective construction of the canopy structure of high-yield wheat.

## Materials and Methods

The whole experiment consists of four small tests (2.1–2.4). The details are as follows.

### Investigating the Effects of Exogenous Hormones on the Occurrence of Wheat Tillers

#### Plant Materials and Experimental Design

Field experiments were carried out from 2014 to 2015 at Experimental Station of Northwest A&F University, Yangling, Shaanxi Province, China (34° 20′ N, 108° 24′ E, 467 m above sea level). The soil was Eum-Orthrosols (Chinese Soil Taxonomy) and maize (*Zea mays* L.) was the previous crop planted in the experimental field. In the 0–30 cm soil layer, the concentration of organic matter was 11.91 g kg^-1^, the total nitrogen was 1.36 g kg^-1^, and the available nitrogen, phosphorus, and potassium in the soil was 54.22, 22.74, and 96.35 mg kg^-1^, respectively. In addition, during the growing seasons, basal fertilizer was applied at the rate of 225 kg N ha^-1^, 75 kg P_2_O_5_ ha^-1^, and 150 kg K_2_O ha^-1^ before planting.

**FIGURE 2:**
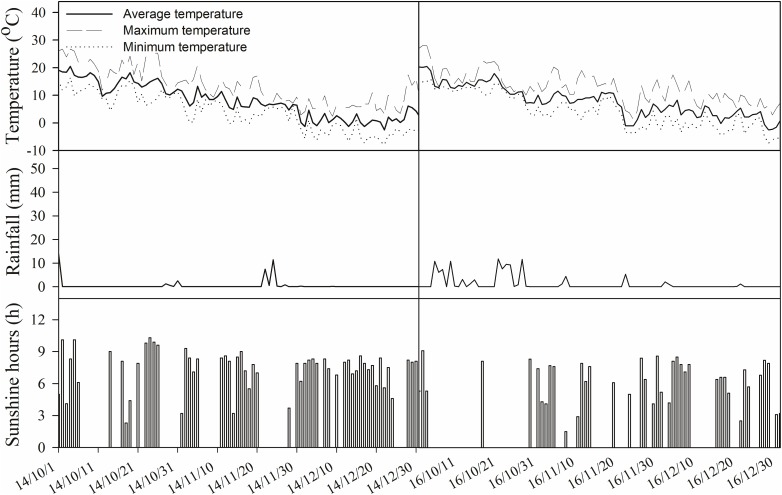
Climate data during the experimental period in the field.

Two typical cultivars of winter wheat (*Triticum aestivum* L.) currently used in local production, Xinong979 and Xiaoyan22, were utilized in this study. Xinong979 (XN979) is a cultivar with a high tiller number and Xiaoyan22 (XY22) is a cultivar with a lower tiller number. The planting densities of the two cultivars were designed with 120 and 90 plants m^-2^, respectively. The experiments followed a completely randomized design consisting of three replications for each cultivar. The plot size was 4 m × 5 m = 20 m^2^ with 20 rows (0.20 m between rows). Climate data collected during the experimental period are shown in Figure 2.

After the wheat seedlings developed four leaves on their main stems, they were divided into the following treatment groups: (1) spraying with deionized water (CK); (2) spraying with 30, 60, and 90 mg L^-1^ IAA (IAA treatment); and (3) spraying with 30, 60, and 90 mg L^-1^ Z (Z treatment).

#### Sampling and Measurements

Thirty days after treatment, uniform plants per plot were selected and the percentage of tiller occurrence at the different tiller positions was investigated. The percentage of tiller occurrence was calculated as the ratio of the number of tillers to total number of plants.

### Investigating the Effects of Exogenous Hormones on the Growth of Wheat Tiller Buds

#### Wheat Growth and Treatments

**FIGURE 3:**
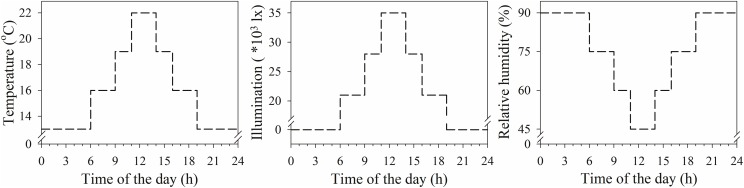
Climate data during the experimental period in plant growth chamber.

The experiments were conducted from 2015 to 2016 in the Agricultural Experiment Laboratory of Northwest A&F University. Seedlings of XN979 and XY22 were planted in plastic garden pots (7 cm × 7 cm × 7 cm, six plants per pot) filled with perlite. Then the plastic pots were transferred to plastic trays (24.5 cm × 25.5 cm × 5 cm, eight pots per plastic tray). The seedlings were cultured with Hoagland’s nutrient solution, which was changed every 24 h. The plants were placed in a growth chamber with conditions simulating those of the typical climate during the wheat tillering stage in the area (Figure 3). When the wheat seedlings developed three leaves on their main stems, they were divided into the following three treatment groups: (1) deionized water was sprayed on the plants (1 mL per plant; CK); (2) 60 mg L^-1^ IAA was sprayed on the plants (1 mL per plant; IAA treatment); and (3) 60 mg L^-1^ Z was sprayed on the plants (1 mL per plant; Z treatment). The concentration of exogenous hormones was determined by preliminary tests, ensuring the normal growth of wheat according to the results presented in a previous study (Liu et al., 2011a).

#### Sampling and Measurements

##### Growth of tiller buds

Sixty tiller buds that were located at the leaf axils of the first leaves (from the bottom) on the main stems were sampled every day for 7 day after the treatments. Sampling consisted of length and weight measurements. Then the buds were divided into groups of three, and the weight of each group was measured.

##### Hormone extraction, purification, and determination

The methods for extraction, purification, and determination of the hormones were carried out as previously described with some modifications (Cao et al., 2006; Zhao et al., 2012; Yang et al., 2014).

###### Hormone extraction

Approximately 0.3 g of tiller nodes and tiller buds were ground with liquid nitrogen, added 4.0 mL of acetonitrile extraction medium containing 30 mg L^-1^ sodium diethyldithiocarbamate as an antioxidant. The homogenate was incubated in the dark at 4°C for 12 h. The extracts were centrifuged at 10000 *g* for 10 min. The residue was further extracted twice with the same solvent.

###### Hormone purification

The supernatant was combined and concentrated to residue under low pressure at 37°C by rotatory evaporation, and re-dissolved in 8.0 mL 0.4 mol L^-1^ phosphate buffer (pH 8.0), and then added 6.0 mL chloroform and oscillated to remove pigment. Chloroform phase was discarded. 0.15 g insoluble polyvinylpyrrolidone was added into the aqueous phase to remove hydroxybenzene, and centrifuged at 10000 *g* for 10 min. 5.0 mL supernatant was pipetted and adjusted to pH 3.0 with pure formic acid. The aqueous phase was extracted by 3.0 mL ethyl acetate two times. The ethyl acetate phase was concentrated by rotatory evaporation under low pressure, and re-dissolved in 1 mL mixed solution, composed of acetonitrile/methanol/0.6% acetic acid solution (5: 50: 45, V: V: V). Finally, the hormone extract was filtered with 0.22 μm hydrophobic membranes.

###### Hormone determination

Analysis of the hormones was carried out using a high performance liquid chromatography (HPLC) system. 20 μL samples was injected into a fixed 20 μL loop for loading onto a Waters symmetry C18 column (4.6 mm × 150 mm, 5 μm). Using acetonitrile: methanol: 0.6% acetic acid solution (5: 50: 45, V: V: V) as mobile phase. Samples were eluted from the column by a Waters series 515 pump at 25°C with a flow rate of 0.6 mL min^-1^. Hormone peaks were detected by a photodiode array detector (Waters 2998 Sparations Module, United States) with the absorbance set at 218, 270, 200, and 262 nm for IAA, Z, GAs, and ABA, respectively.

###### Hormone quantification

The sample hormones were identified by comparing the retention time and characteristic absorption wavelength of different standard hormones (Sigma Chemical, Co., United States). And the amounts of hormones were quantified by comparing the peak area of the samples with those of standard samples. All values were corrected according to the known recovery rates of different hormone concentrations. The recovery rates of Z, IAA, GAs, and ABA were 86.35 ± 3.17, 84.79 ± 3.48, 83.65 ± 4.15, and 88.01 ± 2.98%, respectively (calculating method of the recovery rate: two identical samples were taken, one of which was added with a quantitative standard substance of the component to be tested, and then the two samples were purified and determined simultaneously following the same procedure. The ratio of the difference between the two samples measured values and the added standard substance quantity was the recovery rate.).

### Analyses of the Relationship Between Exogenous Z and the Growth of Wheat Tiller Buds

#### Wheat Plant Growth and Treatments

Plant materials and growth conditions followed those described in the second experiment (2.2). When the wheat seedlings developed three leaves on their main stems, they were divided into the following three treatment groups: (1) deionized water was sprayed on the plants under normal conditions (1 mL per plant; CK); (2) the concentration of NH_4_NO_3_ in Hoagland’s nutrient solution was set as 0.5 mM, and deionized water was sprayed on the plants (1 mL per plant; Low nitrogen level treatment, LN treatment); and (3) the concentration of NH_4_NO_3_ in the Hoagland’s nutrient solution was set as 0.5 mM, and 60 mg L^-1^ Z was sprayed on the plants (1 mL per plant; LN+Z treatment). The concentration of nitrogen used in this experiment was determined by preliminary tests, and the concentrations used were meant to ensure normal wheat growth except for the tiller buds.

#### Sampling and Measurements

##### Growth of tiller buds

The measurement methods were the same as those described in the second experiment (2.2).

##### Hormone extraction, purification, and determination

The measurement methods were the same as those described in the second experiment (2.2).

### Verification of the Effects of Exogenous Z on the Occurrence of Wheat Tillers

#### Plant Materials and Experimental Design

Field experiments were carried out from 2016 to 2017 at the Experimental Station of Northwest A&F University, Yangling, Shaanxi Province, China (34° 20′ N, 108° 24′ E, 467 m above sea level). The soil was Eum-Orthrosols (Chinese Soil Taxonomy) and maize (*Zea mays* L.) was the previous crop. The organic matter concentration was 11.82 g kg^-1^, the total nitrogen was 1.33 g kg^-1^, and the available nitrogen, phosphorus, and potassium concentrations in the soil were 53.58, 22.63, and 98.25 mg kg^-1^ in the 0–30 cm soil layer, respectively. In addition, in growing seasons, basal fertilizer was applied at the rate of 225 kg N ha^-1^ (normal conditions)/22.5 kg N ha^-1^ (low nitrogen conditions), 75 kg P_2_O_5_ ha^-1^, and 150 kg K_2_O ha^-1^ before planting.

XN979 and XY22 were used in the experiment. The experiments were designed as randomized blocks consisting of three replicates for each cultivar. The plot size was 4 m × 5 m = 20 m^2^ with 20 rows (0.20 m between rows). The climate data collected during the experimental period are shown in Figure 2. When the wheat seedlings developed four leaves on their main stems, they were divided into the following three treatment: (1) deionized water was sprayed on the plants under normal conditions (CK); (2) deionized water was sprayed on the plants under low nitrogen conditions (LN treatment); and (3) 60 mg L^-1^ Z was sprayed on the plants under low nitrogen conditions (LN+Z treatment).

#### Sampling and Measurements

The measurement indexes and methods were the same as those described for the first experiment (2.1).

### Statistical Analyses

The results in all of the experiments were analyzed using SPSS 18.0 for Windows, except the principal component analysis (PCA) analysing with Canoco 5. Multiple comparisons were performed after a preliminary *F*-test. The means were tested with a least significant difference test, and the significance was set at the probability level of 0.05.

## Results

### Effects of Exogenous Hormones on the Occurrence of Wheat Tillers

#### Percentage of Tiller Occurrence

Similar trends were observed among the treatments for the two cultivars (Table 1). The occurrence of 0, I, and II were normal under different treatments (data not shown). The percentage of tiller occurrence of III, I-p, IV, I-1, and II-p tended to decline as the IAA application increased, while the differences of percentages between I60 and I90 were not significant. As compared with the CK and IAA treatments, the external application of Z had no effect on the occurrence of III, I-p, IV, I-1, and II-p.

**Table 1:** Percentage of tiller occurrence at the different tiller position under different treatments (%).

Treatment	Tiller position-XN979					Tiller position-XY22				
		
	III	I-p	IV	I-1	II-p	III	I-p	IV	I-1	II-p
CK	96.7 a	100 a	90.0 a	93.3 a	96.7 a	83.3 a	80.0 a	83.3 a	73.3 a	70.0 a
I30	53.3 b	46.7 b	56.7 b	43.3 b	40.0 b	40.0 b	36.7 b	33.3 bc	30.0 c	36.7 b
I60	0.0 c	3.3 c	3.3 c	0.0 c	0.0 c	3.3 d	0.0 d	3.3 d	0.0 d	0.0 d
I90	0.0 c	3.3 c	0.0 c	0.0 c	0.0 c	3.3 d	0.0 d	0.0 d	0.0 d	0.0 d
Z30	100 a	96.7 a	96.7 a	93.3 a	96.7 a	83.3 a	83.3 a	83.3 a	73.3 a	73.3 a
Z60	100 a	100 a	93.3 a	96.7 a	90.0 a	86.7 a	83.3 a	83.3 a	80.0 a	70.0 a
Z90	100 a	100 a	93.3 a	93.3 a	96.7 a	86.7 a	80.0 a	80.0 a	76.7 a	66.7 a


*Values followed by different letters within columns are significantly different at the 0.05 probability level. III: the thirdh primary tiller; I-p: the first secondary tiller from I; IV: the fourth primary tiller; I-1: the second secondary tiller from I; II-p: the secondary tiller from II. CK: deionized water was sprayed on the plants. I30, I60, and I90 denote 30, 60, and 90 mg L^-*1*^ IAA was sprayed on the plants, respectively. Z30, Z60, and Z90 denote 30, 60, and 90 mg L^-*1*^ Z was sprayed on the plants, respectively (*n* = 30).*

#### Growth of Tiller Buds

**FIGURE 4:**
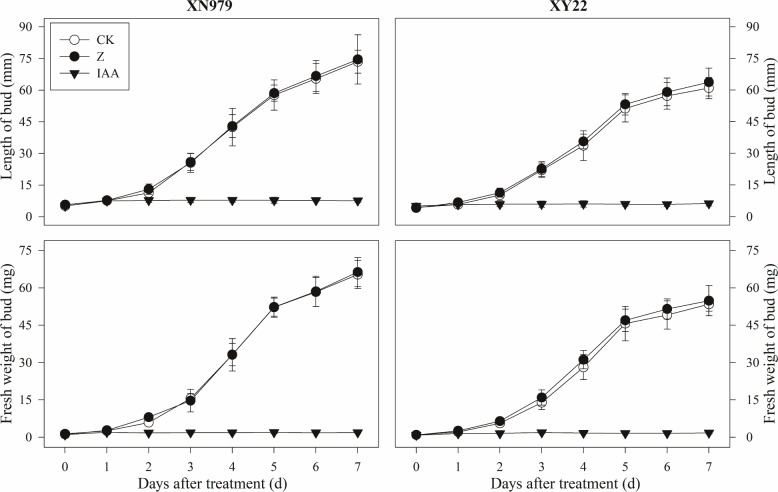
Effects of exogenous hormones on length and fresh weight of tiller buds. CK: deionized water was sprayed on the plants. IAA: 60 mg L^-1^ IAA was sprayed on the plants. Z: 60 mg L^-1^ Z was sprayed on the plants. Segments represent the standard error of the mean (*n* = 30 for length of tiller buds and *n* = 3 for fresh weight of buds).

The same trend was found for the growth of tiller buds in the two cultivars under different treatments. Beginning 1 day after treatment, the length and fresh weight of the tiller buds were significantly greater in the CK group than those in the IAA group (Figure 4). Compared with CK, the external application of IAA completely inhibited the growth of tille buds. The tiller buds grew normally in the Z treatment group, and the length and fresh weight were not significantly different with those in the CK group. However, these measurements were higher than that in the IAA group.

#### Changes in the Hormone Levels

**FIGURE 5:**
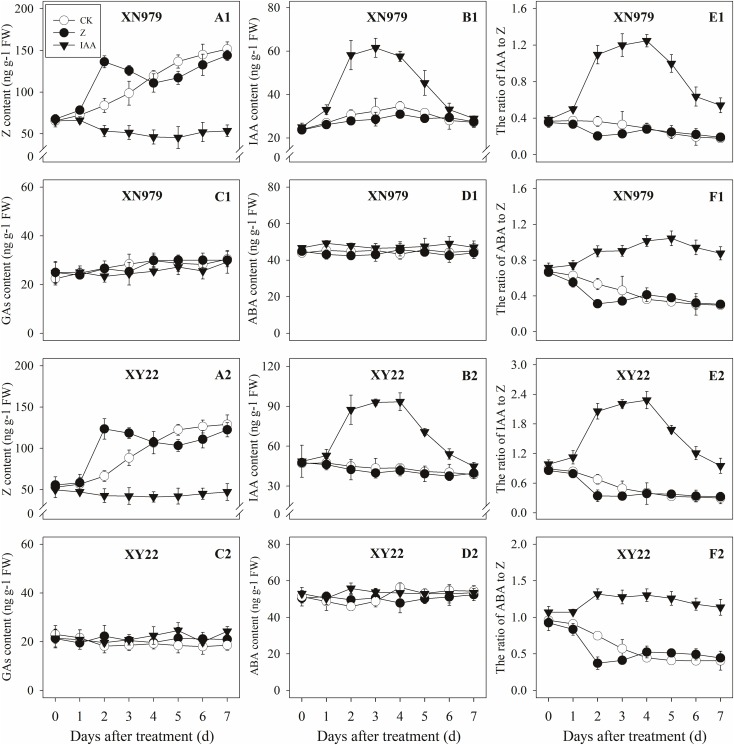
Effect of exogenous hormones on the contents of different hormones in tiller nodes. Panel **(A1–F1)** represent respectively endogenous hormonal (Z, IAA, GAs, ABA, IAA:Z, and ABA:Z) changes in XN979 under different treatments. Panel **(A2–F2)** represent respectively endogenous hormonal (Z, IAA, GAs, ABA, IAA: Z, and ABA:Z) changes in XY22 under different treatments. CK: deionized water was sprayed on the plants. IAA: 60 mg L^-1^ IAA was sprayed on the plants. Z: 60 mg L^-1^ Z was sprayed on the plants. Segments represent ± the standard error of the mean (*n* = 3). FW, fresh weight.

**FIGURE 6:**
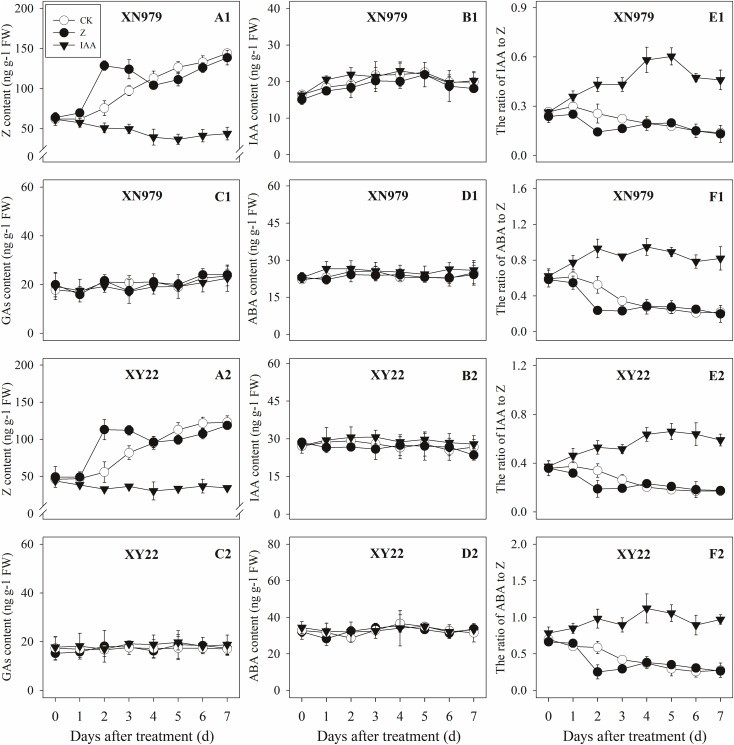
Effect of exogenous hormones on the contents of different hormones in tiller buds. Panel **(A1–F1)** represent respectively endogenous hormonal (Z, IAA, GAs, ABA, IAA:Z, and ABA:Z) changes in XN979 under different treatments. Panel **(A2–F2)** represent respectively endogenous hormonal (Z, IAA, GAs, ABA, IAA: Z, and ABA:Z) changes in XY22 under different treatments. CK: deionized water was sprayed on the plants. IAA: 60 mg L^-1^ IAA was sprayed on the plants. Z: 60 mg L^-1^ Z was sprayed on the plants. Segments represent ± the standard error of the mean (*n* = 3). FW, fresh weight.

The Z content in the tiller nodes increased as the growth of tiller buds progressed. External application of hormones influenced the endogenous Z levels. The Z levels of the Z treatment group increased significantly after treatment, peaking 2 days after treatment then gradually decreasing. From 4 days after treatment, the Z content in the Z treatment had no significant difference compared with the CK while the exogenous application of IAA significantly reduced the Z content (Figures 5A1,A2). Additionally, the dynamics of the Z content and the difference between the treatments were consistent between the tiller buds and tiller nodes (Figures 6A1,A2).

The IAA levels in the tiller nodes remained nearly constant throughout the entire experimental period and were not significantly different among the CK and Z treatment groups. However, the IAA content significantly increased after IAA treatment, peaking at 3 days after treatment and subsequently decreasing gradually. At 7 days after treatment, the IAA content in the IAA treatment was not significantly different compared with the CK and Z treatments (Figures 5B1,B2). Similar to the tiller nodes, the IAA content in the tiller buds remained almost unchanged during the growth of tiller buds. However, the IAA content in the tiller buds did not increase after IAA treatment (Figures 6B1,B2).

Similar to changes in the IAA content, the GAs and ABA levels in the tiller nodes and buds remained nearly constant during the entire experimental period and no significant differences were observed among the CK, IAA, and Z treatment groups (Figures 5C1,C2,D1,D2, 6C1,C2,D1,D2).

The ratio of endogenous IAA to endogenous Z (IAA:Z) gradually declined over the course of the experimental period, and no significant difference was observed in the CK and Z treatment groups. Exogenous IAA significantly increased the IAA:Z levels in the tiller nodes and buds compared with the CK and Z treatment groups; the IAA:Z ratio increased initially, peaking at 4 and 5 days after treatment and then decreased, respectively (Figures 5E1,E2, 6E1,E2).

The ratio of endogenous ABA to endogenous Z (ABA:Z) levels of the CK plants gradually decreased over the course of the experimental period. Exogenous hormones sprayed on plants varied in effect on the ratio of ABA:Z. The ratio remained nearly constant in the Z treatment group after treatment; however, the application of IAA significantly increased the ratio (Figures 5F1,F2, 6F1,F2).

### Effects of Z on the Occurrence of Wheat Tillers Under Low N Conditions

#### Growth of Tiller Buds

**FIGURE 7:**
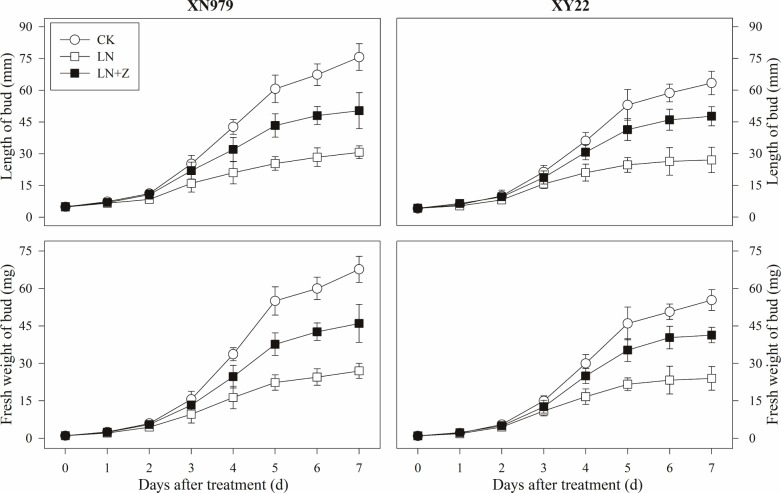
Changes in the length and fresh weight of tiller buds under different treatments. CK: deionized water was sprayed on the plants under normal condition. LN: the concentration of NH_4_NO_3_ in Hoagland’s nutrient solution was set as 0.5 mM, and deionized water was sprayed on the plants. LN+Z: the concentration of NH_4_NO_3_ in Hoagland’s nutrient solution was set as 0.5 mM, and 60 mg L^-1^ Z was sprayed on the plants (*n* = 30 for length of tiller buds and *n* = 3 for fresh weight of buds).

From 2 days after treatment, the length and fresh weight of the tiller buds were significantly greater in the CK group than those in other groups (Figure 7). In the LN treatment group compared with the CK group, slow growth of the tiller buds was observed after 2 days. Spraying with Z significantly promoted the growth of the tiller buds in the low N conditions, in which the length and fresh weight were significantly higher than those in the LN group, but lower than in the CK group from 3 days after treatment.

#### Changes in the Hormone Levels

**FIGURE 8:**
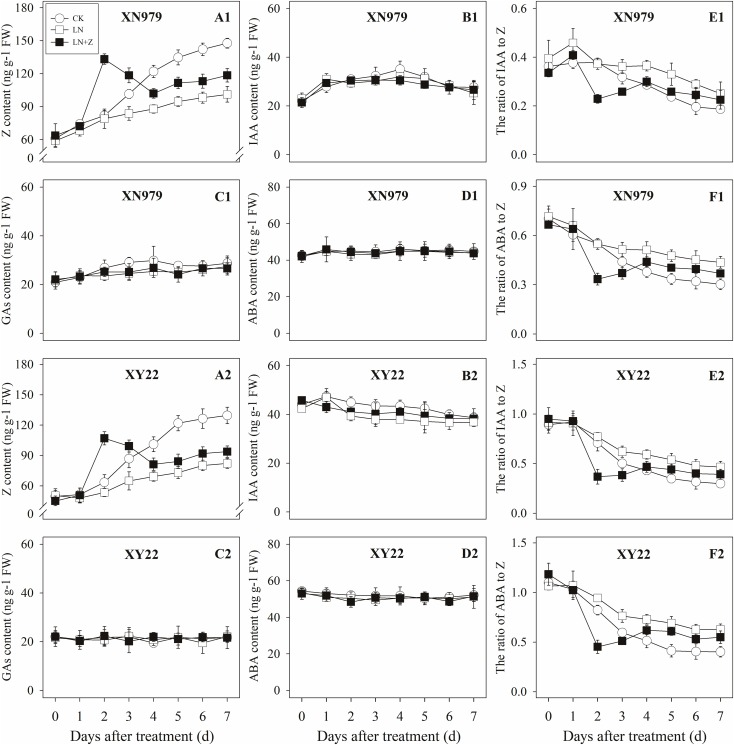
Hormonal changes in tiller nodes under different treatments. Panel **(A1–F1)** represent respectively endogenous hormonal (Z, IAA, GAs, ABA, IAA:Z, and ABA:Z) changes in XN979 under different treatments. Panel **(A2–F2)** represent respectively endogenous hormonal (Z, IAA, GAs, ABA, IAA: Z, and ABA:Z) changes in XY22 under different treatments. CK: deionized water was sprayed on the plants under normal condition. LN: the concentration of NH_4_NO_3_ in Hoagland’s nutrient solution was set as 0.5 mM, and deionized water was sprayed on the plants. LN+Z: the concentration of NH_4_NO_3_ in Hoagland’s nutrient solution was set as 0.5 mM, and 60 mg L^-1^ Z was sprayed on the plants. Segments represent ± the standard error of the mean (*n* = 3). FW, fresh weight.

The Z content in the tiller nodes and buds increased with the growth of the tiller buds under normal conditions, but the levels remained lower than in the control plants under low N conditions. Spraying with Z significantly increased endogenous Z levels after treatment, with a peak observed 2 days after treatment, followed by a gradual decrease in Z levels. From the fourth day after treatment, the Z content was lower than in the plants of the CK treatment groups, but it was significantly higher than the Z content in the plants of the LN treatment groups (Figures 8A1,A2, 9A1,A2).

**FIGURE 9:**
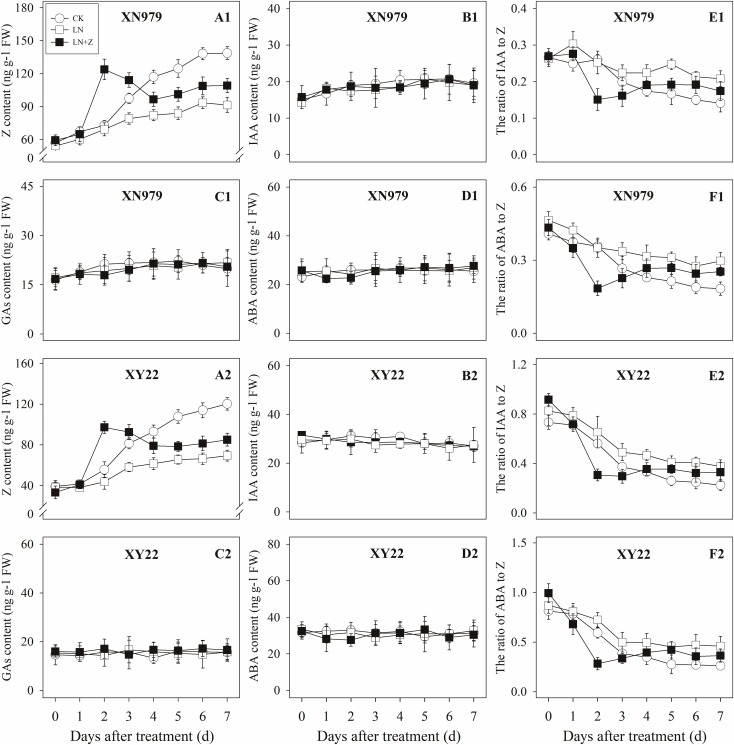
Hormonal changes in tiller buds under different treatments. Panel **(A1–F1)** represent respectively endogenous hormonal (Z, IAA, GAs, ABA, IAA:Z, and ABA:Z) changes in XN979 under different treatments. Panel **(A2–F2)** represent respectively endogenous hormonal (Z, IAA, GAs, ABA, IAA: Z, and ABA:Z) changes in XY22 under different treatments. CK: deionized water was sprayed on the plants under normal condition. LN: the concentration of NH_4_NO_3_ in Hoagland’s nutrient solution was set as 0.5 mM, and deionized water was sprayed on the plants. LN+Z: the concentration of NH_4_NO_3_ in Hoagland’s nutrient solution was set as 0.5 mM, and 60 mg L^-1^ Z was sprayed on the plants. Segments represent ± the standard error of the mean (*n* = 3). FW, fresh weight.

The IAA, GAs, and ABA levels in the tiller nodes and buds remained nearly constant throughout the entire experimental period and were not significantly different among the CK, LN, and LN+Z treatment groups (Figures 8, 9).

The IAA:Z levels of the control plants gradually decreased throughout the experimental period. Compared with the control, plants grown in low N conditions had a high level of IAA:Z. The external application of Z on the plants in the LN treatments significantly reduced the IAA:Z levels after treatment, with a valley observed 2 days after treatment followed by a gradual increase. From the fourth day after treatment, the ratio of IAA to Z was higher than that of the CK treatment groups, but IAA:Z levels was significantly lower than the ratio observed for the LN treatment groups (Figures 8E1,E2, 9E1,E2).

Similar to changes in the IAA:Z levels, the ratio of ABA to Z in the tiller nodes and buds gradually decreased throughout the experimental period. The external application of Z on plants in the LN groups significantly reduced the ABA:Z levels initially, followed by a gradual increase (Figures 8F1,F2, 9F1,F2).

#### Percentage of Tiller Occurrence

The occurrence of 0, I, and II were normal under different treatments (data not shown). Compared with normal conditions, the percentage of tiller occurrence of III, I-p, IV, I-1, and II-p markedly decreased in the LN treatment group. The application of Z increased the percentage of occurrence of III, I-p, IV, I-1, and II-p under low N conditions in the two cultivars. Though the application of exogenous Z increased the occurrence of wheat tillers in most treatments, this effect was significantly reduced with the shifting of tiller position from low to high (Table 2).

**Table 2:** Effect of exogenous Z on the occurrence of wheat tiller under two different N environments (%).

Treatment	Tiller position-XN979					Tiller position-XY22				
		
	III	I-p	IV	I-1	II-p	III	I-p	IV	I-1	II-p
CK	100 a	96.7 a	96.7 a	93.3 a	90.0 a	83.3 a	80.0 a	83.3 a	73.3 a	70.0 a
LN	53.3 c	56.7 c	33.3 c	30.0 c	30.0 c	50.0 c	46.7 c	33.3 c	30.0 c	36.7 c
LN-Z	93.3 a	90.0 a	76.7 b	66.7 b	70.0 b	80.0 a	73.3 a	53.3 b	56.7 b	50.0 b


*Values followed by different letters within columns are significantly different at the 0.05 probability level. III: the thirdh primary tiller; I-p: the first secondary tiller from I; IV: the fourth primary tiller; I-1: the second secondary tiller from I; II-p: the secondary tiller from II. CK: deionized water was sprayed on the plants under normal condition. LN: basal fertilizer was applied at the rate of 22.5 kg N ha^-*1*^, and deionized water was sprayed on the plants. LN+Z: basal fertilizer was applied at the rate of 22.5 kg N ha^-*1*^, and 60 mg L^-*1*^ Z was sprayed on the plants (*n* = 30).*

### Relationship Between Endogenous Hormones and the Growth of Tiller Buds

#### Principal Component Analysis

**FIGURE 10:**
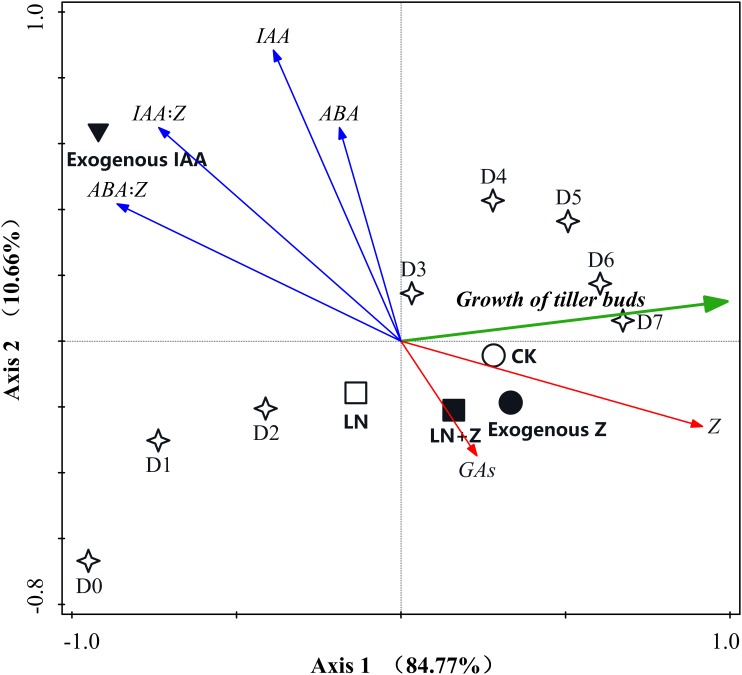
Principal component analysis (PCA) two-dimensional ordination diagram between the growth of wheat tiller buds and endogenous hormones under different treatments. D1–D7: days after treatment.

Principal component analyses were conducted to reduce the set of hormone variables involved in the growth of tiller buds and to extract a small number of latent factors for analyzing the relationship among the observed variables. The results of the PCA analysis showed that the six variables were classified into three groups: (1) the growth of the tiller buds clustered in the first quadrant; (2) Z and GAs clustered in the second quadrant; and (3) ABA, IAA, IAA:Z, and ABA:Z were in the fourth quadrant. In the two-dimensional PCA ordination diagram, the small angle between arrows indicates a high correlation between variables, and arrows pointing in opposite directions indicate a negative correlation in the space of the two principal components. Our results indicated that the growth of the tiller buds showed a high positive correlation with Z and GAs, suggesting that Z and GAs are the positive regulators involved in promoting the growth of tiller buds. Moreover, the cosine value of the angle between tiller bud growth and Z was greater than that between tiller bud growth and GAs, indicating that Z plays a large role in the growth of tiller buds than GAs. The growth of tiller buds showed a highly opposite correlation with ABA, IAA, IAA:Z, and ABA:Z, indicating that these hormones acted as negative regulators inhibiting tiller bud growth; the regulating effect showed the following trend: ABA:Z > IAA:Z > IAA > ABA (Figure 10).

#### Correlation Analysis

**FIGURE 11:**
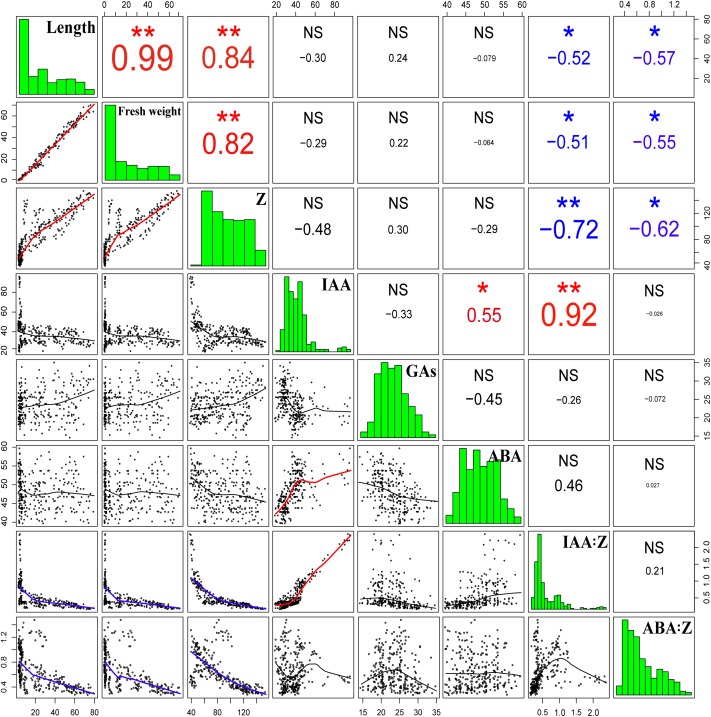
Correlation coefficients of endogenous hormone contents with the lengths and fresh weights of tiller buds. Correlation coefficients (r) are calculated and asterisks (^∗∗^) represent significance at the 0.01 probability level and asterisks (^∗^) represent significance at the 0.05 probability level (*n* = 288).

The correlation analysis demonstrated that the length and fresh weight of tiller buds were positively significantly correlated with the Z content and negatively significantly correlated with the ratio of IAA to Z and ABA to Z. In addition, the growth of tiller buds showed no significant correlations with the level of IAA, GAs, and ABA (Figure 11).

## Discussion

### Effect of Exogenous Hormones on the Occurrence of Wheat Tillers

The occurrence of wheat tillers is regulated by endogenous and environmental cues. In addition to genetics (Xie et al., 2016) and nutrition (Sakakibara et al., 2006; Mason et al., 2014), hormones play key roles in the development of wheat tillering, and the external application of hormones can regulate the occurrence and growth of tillers (Chatfield et al., 2000; Liu et al., 2011b). The growth of target tillers regulated by exogenous hormones is closely related to the leaf age of crops at the time of treatment (Wang et al., 2002). In the present study, the occurrence of 0, I, and II were normal under different treatments because of the treatment time. The percentage of occurrence of III, I-p, IV, I-1, and II-p tended to decline as the application of IAA increased. Compared with the CK treatment, the exogenous application of Z had no obvious effect on promoting the occurrence of tillers.

The tillers develop from tiller buds. Further analysis of the effects of exogenous hormones on the growth of tiller buds shows that spraying IAA completely inhibited the growth of tiller buds, which is similar to the findings described by a previous study (Liu et al., 2011a). But in the present study, exogenous Z treatment had no obvious effect on the growth of tiller buds. The growth of tiller buds is directly related to endogenous hormones (Lin et al., 2016). Therefore, we measured the hormone dynamics throughout an entire experimental period, and found that the content of endogenous Z increased as the growth of tiller buds progressed. External IAA application significantly reduced the Z content compared with the control, while the Z levels increased significantly after the application of exogenous Z in a short period but then no significant difference was observed in the CK and Z treatment groups. Therefore, we speculate that exogenous Z does not promote the growth of tiller buds because the external application of Z has no significant effect on endogenous Z.

Previous studies have demonstrated that nitrogen is a substrate for CTK synthesis, thus regulating the concentration of nitrogen in the environment can change the level of endogenous CTK (Yang et al., 2017). Based on this finding, we also conducted a nitrogen experiment to further analyze the role of Z in regulating the growth of tiller buds under two different N environments. The results showed that the endogenous Z content of plants in the low-nitrogen treatment group was significantly lower than that in the CK group, and the growth rate of tiller buds also showed a significant slowing of growth. In addition, the application of Z on the plants in the low-nitrogen treatment showed a significant increase in the endogenous Z content. Compared with the low-nitrogen treatment group, the growth rate of tiller buds of the LN+Z group was significantly accelerated. This indicates that exogenous Z can effectively promote the growth of tiller buds in an environment low in nitrogen. Subsequently, we set up a low-nitrogen treatment in the field, verifying that exogenous Z can significantly increase the survival rate of high position tillers under low N conditions, and thus promote the occurrence of wheat tillers.

Accordingly, we conclude that plant hormones can effectively control wheat tiller occurrence, and the application of exogenous hormones changes the growth of tiller buds via affecting the endogenous hormone content, thus regulating the occurrence of tillers in wheat. Exogenous IAA could inhibit the occurrence of tillers and reduce the quantity of wheat population; exogenous Z can increase the survival rate of tillers when the growth of tiller buds is affected by biotic and abiotic stressors, thereby increasing the population.

### Relationship Between Endogenous Hormones and the Growth of Tiller Buds

External factors influence wheat tillering through physiological effects, which are predominantly mediated by the variations in hormone content (Kariali and Mohapatra, 2007). IAA and CTK play important roles in regulating the growth of plant lateral buds (Wang et al., 2006; Xu et al., 2015). Specifically, IAA inhibits the tillering process by producing apical dominance (Leopold, 1949). Liu et al. (2011a) also reported that the application of NAA completely inhibited the growth of rice tiller buds. Our data clearly demonstrated that exogenous application of IAA completely inhibited the growth of wheat tiller buds, thus our findings are consonant with previous studies. However, some experiments have shown that the application of exogenous IAA to the top of the plant does not enter the lateral buds (Booker et al., 2003). In this experiment, the external application of IAA significantly increased the content of endogenous IAA in the tiller node but had no obvious effect on the IAA content in the tiller buds. Also, reducing the auxin content in the stem via the application of growth polar transport inhibitors does not promote the occurrence of lateral buds (Morris et al., 2005). Furthermore, according to the PCA and correlation analysis, no significant correlation was observed between the growth of tiller buds and the level of endogenous IAA, indicating that IAA does not directly inhibit the growth of lateral buds, and there may be a secondary messenger that transmits IAA signals to the lateral buds. A previous study has suggested that the second messenger has CTKs, etc. (Domagalska and Leyser, 2011).

Cytokinin promotes cell division and plays a key role in the growth and differentiation of plant cells and tissues. Through the phenotypic analysis of the mutants of the CTK signaling receptor system, a previous study has found that overexpressing CTK in the recessive mutant has a multi-branched phenotype (Manuella et al., 2002) and the petunia mutant also showed multiple branches through enhancing the expression of an isoamyl transferase gene (synthesis of active CTK) (Elena et al., 2002). In addition, higher CTK content can promote the germination of corn tiller buds (Wang et al., 2012a) and the growth of rice tiller buds (Liu et al., 2012). In this experiment, endogenous CTK showed an upward trend during the normal growth of wheat tiller buds. By reducing the nitrogen concentration of the nutrient solution to reduce the endogenous CTK in the plants, the growth rate of the tiller buds slowed, and this rate could be adjusted by exogenous application of CTK to change the endogenous CTK content. All the above research has demonstrated that CTK directly promotes the growth of tiller buds.

Many studies have demonstrated that the synthesis and accumulation of CTK are regulated by IAA; in addition, as an upstream signaling hormone, IAA inhibits the synthesis of CTK in plant tissues (roots, stems, etc.) and its translocation to other organs (Nordström et al., 2004; Tanaka et al., 2006). In the present study, we also found that after the application of exogenous IAA, the endogenous Z content decreased during the growth of tiller buds and was lower than that of plants grown under normal conditions. Based on the above results, we conclude that endogenous Z plays a leading role in the regulation of the growth of wheat tiller buds, while the effects of endogenous IAA are more indirect. Exogenous IAA regulates the accumulation of endogenous Z by affecting the distribution and quantity of IAA in the plant, and then inhibiting the growth of the tiller buds.

In addition to the effects of IAA and CTK on tillering, there are many reports on the relationship between GAs and tillering. Spraying GA_3_ can significantly inhibit the occurrence of crop tillers (Zhang, 1997). Some studies have speculated that exogenous GA_3_ inhibits the growth of tiller buds by affecting plant nutrition (Zhang, 2006), but Liu et al. (2011a) pointed out that the growth of tiller buds is not necessarily related to carbon and nitrogen metabolism in plants, and the buds growth is mainly regulated by hormone metabolism. In this experiment, the PCA and correlation analysis indicated that there was no significant correlation between endogenous GAs and tiller growth. Phillips (1975) suggested that GAs indirectly affects apical dominance by inducing IAA synthesis or inhibiting IAA decomposition. Cai et al. (2013) further found that exogenous GA_3_ affects the growth of tiller buds by changing the endogenous ratios of IAA to Z and ABA to Z. Furthermore, ABA is closely related to lateral bud dormancy (Pei et al., 1998). Some studies have shown that the higher ABA content in the regenerated buds of rice is the main factor inhibiting the buds growth (Wang et al., 1997), and the increase in endogenous ABA content of wheat tillers inhibits the further development of tillers and leads to decline (Ma and Liang, 1997).

However, Liu et al. (2011a) concluded that there was no significant correlation between ABA and the growth of tiller buds in rice; our previous study also found that spraying ABA did not completely inhibit the growth of wheat tiller buds, but only slowed its growth rate, indicating that ABA is not the main factor regulating the growth of tiller buds. However, some results have revealed that the application of ABA significantly increased the ratio of ABA to Z, while higher ABA:Z inhibited the growth of tiller buds and promoted the decline of tillering (Xu et al., 2010; Wang et al., 2012a). Therefore, ABA mainly influenced the growth of tiller buds via the balance of ABA to Z. Additionally, a few studies have indicated that the mechanisms of axillary bud outgrowth depend on the relative content of endogenous hormones rather than the absolute level of an individual hormone (Shimizu-Sato and Mori, 2001; Luo et al., 2014). The data from the present study agreed with these results in that the ratios of IAA to Z and ABA to Z were significantly negatively correlated with the growth of wheat tiller buds, indicating that the ratios had an essential role in the growth of tiller buds. Nevertheless, our data also showed that the endogenous Z content increased with the growth of tiller buds, while the IAA and ABA levels remained nearly constant during the entire experimental period. These results suggest that the difference in IAA:Z and ABA:Z was more due to the variation of the Z content. Therefore, we can conclude that exogenous hormones affected the accumulation of endogenous Z in the tiller nodes and buds through altering the synthesis and distribution of endogenous hormones, thus regulating the growth of wheat tiller buds.

However, at this stage, the mechanism by which hormones regulate the growth of wheat tiller buds is understood at the physiological and biochemical level, but research is lacking at the molecular level. Recently, some new plant hormones have been discovered, such as strigolactone (Umehara et al., 2008), but the role of these hormones in the growth of wheat tiller buds and how these can be applied to the control of wheat tiller occurrence are currently poorly understood. Therefore, these issues need to be further studied and clarified.

## Conclusion

**FIGURE 12:**
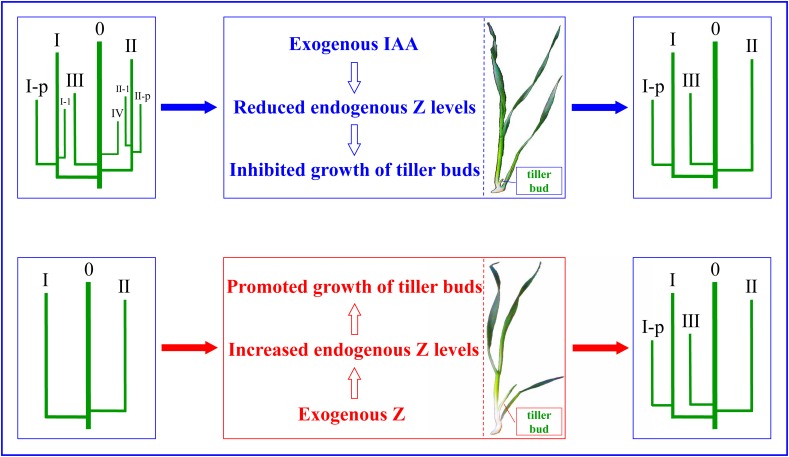
The regulatory mechanism of exogenous IAA and Z on the occurrence of wheat tiller.

We proposed and verified a technical approach to effectively regulate the growth of wheat tillers: when the number of wheat population grew too large, the number can be reduced by applying IAA to inhibit the occurrence of tillers; and when the population number was less than the expected value, exogenous Z can be sprayed to promote tillering so as to increase the number. Multiple hormones regulate the growth of wheat tillers. For the different opinions on the role of endogenous hormones in the growth of tiller buds at present, the following results have been obtained through experiments. Exogenous IAA completely inhibited the growth of tiller buds through reducing endogenous Z levels. External application of Z increased the endogenous Z content, thus promoting the growth of the wheat tiller buds. The PCA and correlation analysis demonstrated that the growth of the tiller buds was significantly positively correlated with the content of Z, suggesting that Z plays key roles in regulating the occurrence of tillers, and exogenous hormones regulated the growth of wheat tiller buds via affecting endogenous Z levels, thus regulating the occurrence of tillers in wheat (Figure 12).

## Author Contributions

DY, XR, and TC conceived and designed the experiments. TC, XM, and XL performed the experiments. XL and TL analyzed the data. TC, XM, and XL wrote the paper. HW, ZJ, XR, and DY reviewed and revised the paper. TL and ZJ corrected the English language for the paper. The manuscript was reviewed and approved for publication by all authors.

## Conflict of Interest Statement

The authors declare that the research was conducted in the absence of any commercial or financial relationships that could be construed as a potential conflict of interest.
